# Circulating soluble apo 2L, soluble OX-2: a possible screening biomarker for stage-4 ovarian, endometrial CA

**DOI:** 10.1186/1939-4551-8-S1-A12

**Published:** 2015-04-08

**Authors:** Arzu Didem Yalcin, Betul Celik, Selahattin Kumru, Saadet Gumuslu

**Affiliations:** 1 Internal Medicine, Allergy and Clinical Immunology, Genomics Research Center, Academia Sinica,11529, Taipei, Taiwan; 2 Department of Laboratory Medicine and Pathology, Mayo Clinic in Jacksonville, USA; 3 Antalya Education and Research Hospital, Turkey; 4 Department of Medical Biochemistry, Faculty of Medicine, Akdeniz University, 07070, Antalya, Turkey

## Background

The aim of this study is to evaluate soluble tumor necrosis factor-related apoptosis inducing ligand (sApo 2L, sTRAIL) levels in ascites fluid to predict its clinical usage in detecting malignant ascites and soluble CD200 (sCD200, sOX-2) levels to predict its clinical usage in blood detecting breast cancer.

## Methods

Ascites and sera samples from patients without known malignancy at the admission were collected. There were 14 stage-4 breast cancer (BC), 17 stage-4 ovarian cancer (OC) and 19 stage-4 endometrial cancer (EC) diagnosed later on. Control groups consisted of benign peritoneal fluids (n=53) and sera samples (n=25) from healthy subjects.

Concentrations of sCD200 in the serum samples were quantified using ELISA kit. CEA (Beckman Coulter System. Catalog Number:33200), CA-19.9(Beckman Coulter System. Catalog Number:387687), CA-125 (Beckman Coulter System. Catalog Number:386357) and CA15.3 (Beckman Coulter System. Catalog Number: 387620). Levels were enumerated by fluoroenzyme immunoassay. Concentrations of sTRAIL in the serum samples were quantified using ELISA kits (Diaclone, France).

## Results

The significant low level of sApo 2L was observed in peritoneal fluids from OC and EC patients than benign peritoneal fluids from control patients. Besides, positive correlation was observed between sApo 2L and aspartate aminotransferase (AST) in benign peritoneal fluid and sOX-2 and creatinine and sOX-2 and platelet in OC patients and sOX-2 and carcinoembrionic antigen (CEA) in EC patients and sOX-2 and blood urea nitrogen (BUN) in healthy subjects.

## Conclusions

Our data indicate that low level of sApo 2L is a good biochemical marker detecting malignant ascites. Further decline in the level of sApo 2L was seen in EC than OC. Since higher level of sApo 2L was seen in higher level of AST, liver might involved its metabolism. Positive correlation detected between sOX-2 and creatinine, platelet, CEA, BUN needs to be elucidated.

